# Supplementary material for: Near-Infrared In Vivo Imaging of Claudin-1 Expression by Orthotopically Implanted Patient-Derived Colonic Adenoma Organoids
Source: Diagnostics (Basel). 2024 Jan 26;14(3):273. doi: 10.3390/diagnostics14030273 (PMC10854921; doi:10.3390/diagnostics14030273)
Supplement: Supplementary file 1 [file diagnostics-14-00273-s001.zip › Figures S1-S3.pdf]

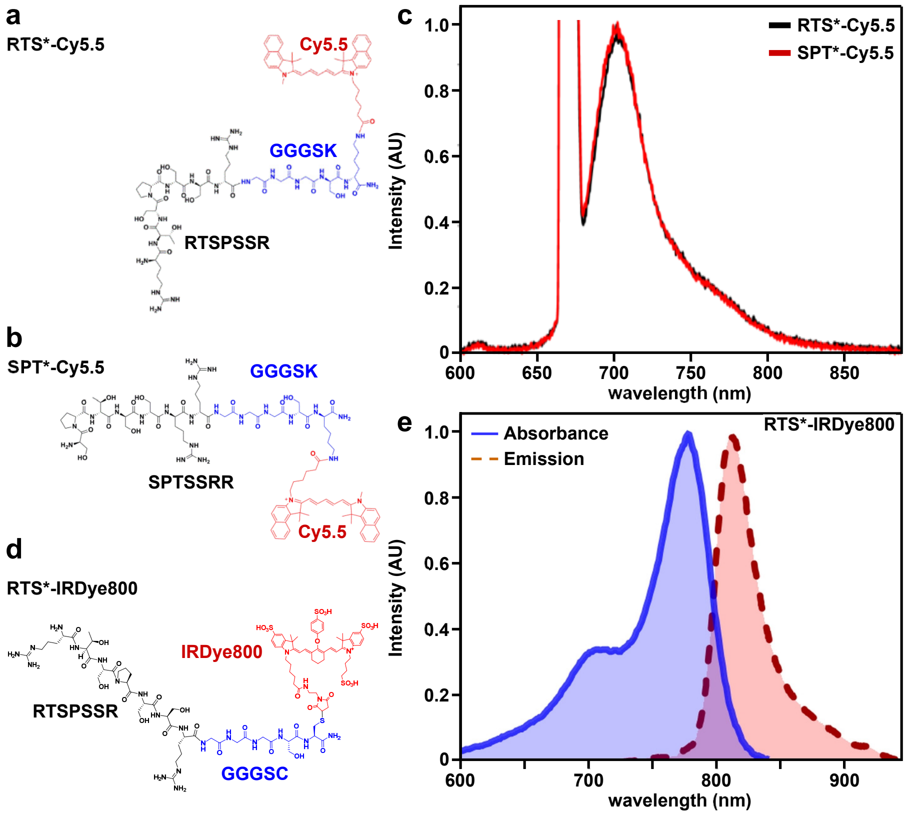

Figure S1. Peptides specific for claudin-1. (**a**) RTSPSSR was labeled with IRDye800CW (red) via a GGGSC linker (blue), and (**b**) with Cy5.5 (red) via a GGGSK linker (blue) to prevent steric hindrance. (**c**) SPTSSRR (control) was labeled with Cy5.5 (red) via a GGGSK linker (blue). (**d**) Peak absorbance and fluorescence emission wavelengths for RTS*-IRDye800 were identified at λ_{abs} = 785 nm and λ_{em} = 812 nm, respectively. (**e**) Fluorescence emission spectra for RTS*-Cy5.5 and SPT*-Cy5.5 peaked at λ_{em} = 710 nm. Peak absorbance and fluorescence emission wavelengths in the NIR spectrum minimize tissue autofluorescence, hemoglobin absorption, and tissue scattering.

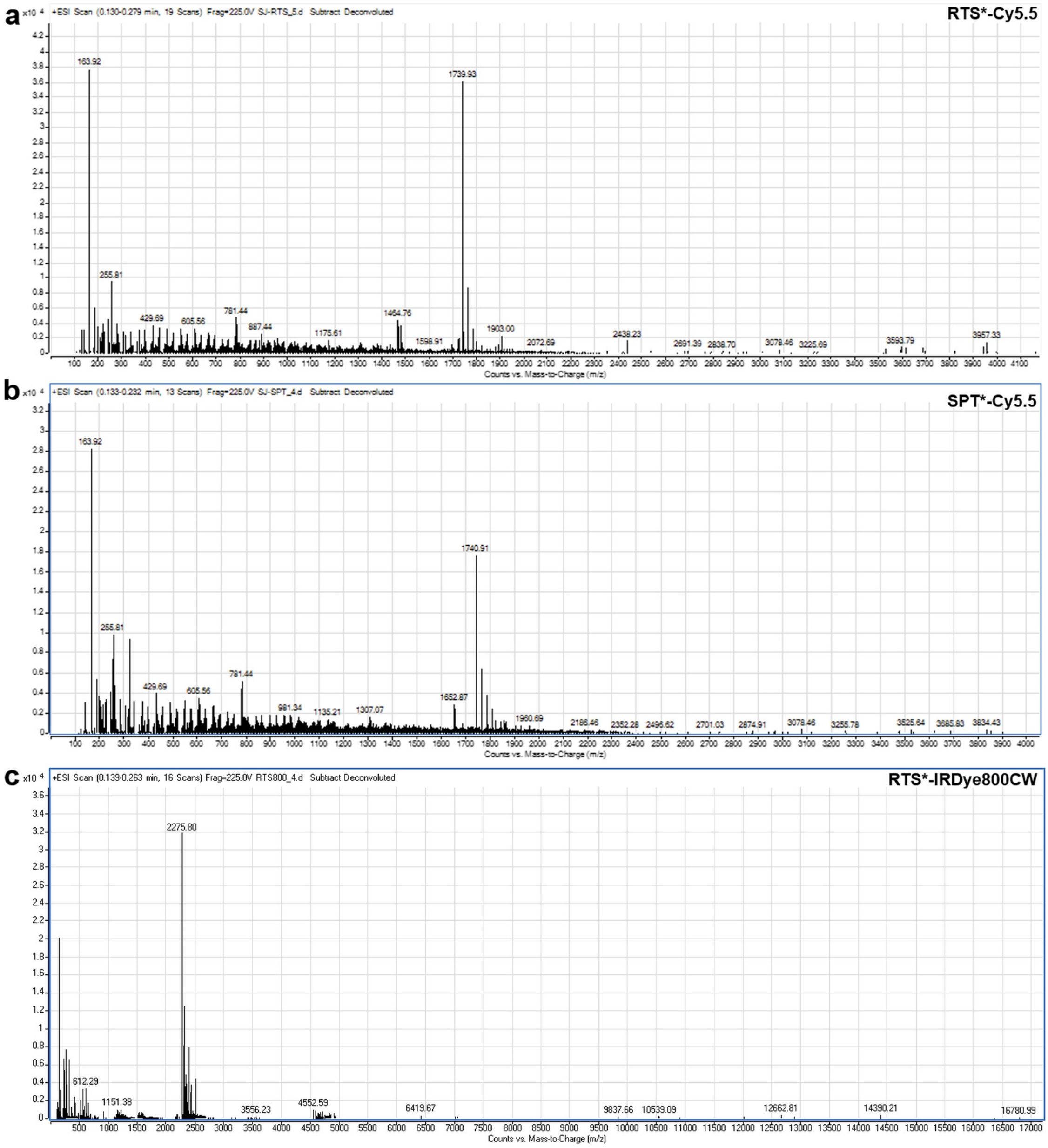

Figure S2. Mass spectrometry. (**a**) A mass-to-charge (m/z) ratio of 2275.80 was measured for RTS*-IRDye800CW, which agreed with the expected value of 2275.81. A mass-to-charge (m/z) ratio of 1739.93 and 1740.91 were measured for (**b**) RTS*-Cy5.5 and (**c**) SPT*-Cy5.5, respectively, which agreed with the expected values.

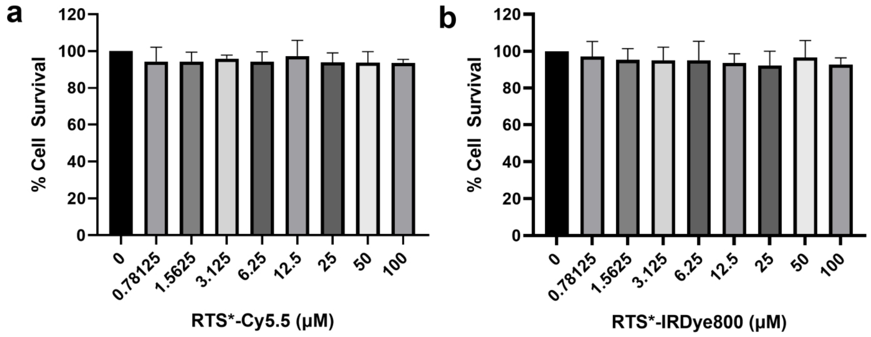

Figure S3. Peptide cytotoxicity to normal cells. MTT assay showed no effect on survival with normal human colon cells (CCD 841 CoN) following treatment with (a) RTS*-Cy5.5 and (b) RTS*-IRDye800, $P = 0.88$ and $P = 0.93$, respectively, using a one-way ANOVA model.
